# Effects of an air pollution personal alert system on health service usage in a high-risk general population: a quasi-experimental study using linked data

**DOI:** 10.1136/jech-2016-207222

**Published:** 2016-05-23

**Authors:** R A Lyons, S E Rodgers, S Thomas, R Bailey, H Brunt, D Thayer, J Bidmead, B A Evans, P Harold, M Hooper, H Snooks

**Affiliations:** 1 Swansea University Medical School, Swansea, UK; 2 Cwm Taf Public Health Team, Public Health Wales, Keir Hardie University Health Park, Merthyr Tydfil, UK; 3 Health Protection Team, Public Health Wales, Cardiff, UK; 4 Member of the Public, Swansea, UK; 5 Public Health England, Centre for Radiation Chemical and Environmental Hazards (Wales), Metropolitan University, Cardiff, UK; 6 Neath Port Talbot County Borough Council, Neath, UK

**Keywords:** AIR POLLUTION, HEALTH SERVICES, RECORD LINKAGE

## Abstract

**Background:**

There is no evidence to date on whether an intervention alerting people to high levels of pollution is effective in reducing health service utilisation. We evaluated alert accuracy and the effect of a targeted personal air pollution alert system, airAware, on emergency hospital admissions, emergency department attendances, general practitioner contacts and prescribed medications.

**Methods:**

Quasi-experimental study describing accuracy of alerts compared with pollution triggers; and comparing relative changes in healthcare utilisation in the intervention group to those who did not sign-up. Participants were people diagnosed with asthma, chronic obstructive pulmonary disease (COPD) or coronary heart disease, resident in an industrial area of south Wales and registered patients at 1 of 4 general practices. Longitudinal anonymised record linked data were modelled for participants and non-participants, adjusting for differences between groups.

**Results:**

During the 2-year intervention period alerts were correctly issued on 208 of 248 occasions; sensitivity was 83.9% (95% CI 78.8% to 87.9%) and specificity 99.5% (95% CI 99.3% to 99.6%). The intervention was associated with a 4-fold increase in admissions for respiratory conditions (incidence rate ratio (IRR) 3.97; 95% CI 1.59 to 9.93) and a near doubling of emergency department attendance (IRR=1.89; 95% CI 1.34 to 2.68).

**Conclusions:**

The intervention was associated with increased emergency admissions for respiratory conditions. While findings may be context specific, evidence from this evaluation questions the benefits of implementing near real-time personal pollution alert systems for high-risk individuals.

## Introduction

### Background

The UK Government has responded to the growing evidence of adverse health effects from air pollution by specifying human health protection exposure limits.^1^ These recommend implementing effective measures to reduce pollution levels and minimise public exposure and consequent impacts.^2–4^ Recommendations made by the Committee on the Medical Aspects of Air Pollution (COMEAP) in 2011 stimulated the development of air quality alert systems providing important information and advice to the public to minimise air pollutant exposure.^5–8^ These are expected to reduce adverse effects of pollution and health service utilisation through reduction of symptoms. Air quality alert systems have operated in the UK for a number of years, including the airText system in London, airAlert in Sussex, Know and Respond in Scotland and the Met Office has Healthy Outlook. All operate on forecasts of air quality for the following day.^9–12^ We only found one published quantitative evaluations of an air quality alert system. This evaluation assessed changes in additional hospital admissions estimated for a test group of patients who would be likely to sign-up for the alert service, modelled against the current level of admissions for the populations of London and Sussex, UK.^13^

The airAware system was a novel development because it integrated near real-time data rather than forecasting. Its design facilitated early identification of local air pollution problems and issued timely warnings of air pollution episodes reflecting levels of particulate matter (measured as particulate matter 10 μm or less in diameter (PM_10_)) at nearby air quality monitoring stations. People who signed up to the system received alerts from an independent contractor who operated the airAware system on behalf of the multiagency Local Service Board (who had responsibility to oversee the delivery of the project). The contractor managed participant sign-up and the issue of alerts.

The airAware system operated in a setting with a well-established population data linkage system capable of supporting the evaluation of individual-level interventions.^14–16^ We undertook a retrospective cohort study using individual linked data to evaluate the effectiveness and impact of this novel system.

### Objectives

Our objectives were: (1) to evaluate the accuracy of the airAware system in correctly issuing alerts in terms of sensitivity, specificity, positive and negative predictive values (NPVs); and (2) to evaluate the effect of the system on subsequent health service utilisation. We have reported this evalution following STROBE guidelines.

## Methods

### Study design

We created an electronic cohort of individuals invited to sign-up to the system from anonymised primary care data to evaluate the impact of the air quality alerting system for patients with pollution-susceptible diseases on their health outcomes, recorded in routinely collected data. The invitation, patient information leaflet and patient registration welcome pack are shown in the online supplementary material. We compared changes in primary and secondary care health service utilisation preintervention and postintervention for an intervention group who signed up to the alert system, to the control group who did not.

10.1136/jech-2016-207222.supp1 Supplementary data

### Setting

An Air Quality Management Area (AQMA) in Port Talbot, an industrial urban area of south Wales, UK, adjacent to a steelworks and motorway, with six air pollution monitors for PM_10_ particulates.

### Intervention

**Figure 1:**
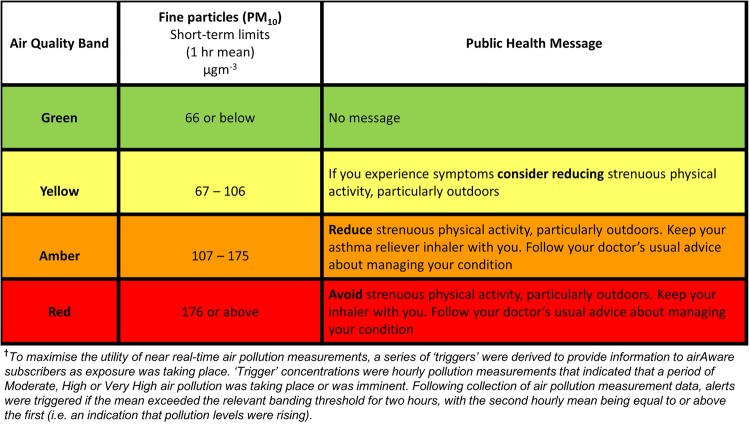
Air quality bands, alert trigger† threshold criteria and health messages.

The airAware system sent alerts in near real-time by text, email or pre-recorded voice. Alerts, automatically triggered by higher pollution levels, advised of changed air quality and used advice on self-care and healthy behaviour. Messages were based on COMEAP Air Quality Index Health Advice^5^ and agreed by the project team (academics, public health practitioners, respiratory physicians, general practitioners (GPs), technical staff and lay members) and a service user group called SUCCESS (Service Users with Chronic Conditions Encouraging Sensible Solutions) to ensure they could be clearly understood by participants. Alert messages and trigger criteria are shown in figure 1. To avoid overalerting, messages were sent between 7:00 and 22:00 only. Once air quality returned to normal levels, an ‘all-clear’ message was sent. The number of messages per day was limited to three; on any single day no more than three alerts could be issued. Only one alert was issued for the day unless a higher pollution trigger level was met during the daily alerting period.

### Participants

Eligible participants were patients registered with one of four local general practices, with asthma, chronic obstructive pulmonary disease (COPD) or coronary heart disease (CHD). They were included on the practice registers for these conditions as required by the Quality and Outcomes Framework (QOF) of the National Health Service (NHS) General Medical Services contract. The list of General Practice Read codes defining these conditions can be found in the the QOF.^17^

Patients meeting the inclusion criteria were individually invited by GP letter in May 2012 to sign-up to the airAware system. We used a QOF-based algorithm to generate invitees, the QOF is an incentive-based programme for GPs based in the UK to assess their achievements, for example, in regularly treating diagnosed conditions.^17^ The letter was accompanied by a pack that included an information leaflet (with online registration details), paper registration sheet and prepaid addressed return envelope. All documentation was produced in English and Welsh. The leaflet and pack were developed by the project team that included service users. The invitation letter and information leaflet, and registration pack are shown in the online supplementary files.

To register, participants selected: preferred method of message receipt (voice message to land-line, text to mobile phone, or email); preferred language (English or Welsh); from which of the six local monitors (identified by name and location) they wished to receive alerts. Text message was the most popular method of receiving the messages (43%), followed by voice message (31%) and email (26%). Intervention and control group participants were flagged in an anonymised databank for follow-up (the Secure Anonymised Information Linkage (SAIL) databank).^14^ ^15^

### System performance

#### Data sources

Air pollution data were routinely obtained from six local authority-operated monitors. Hourly air pollution data, measured at each monitoring station, were received by the system contractor and used to forecast potential 24-hour episodes for PM_10_.

#### Variables

We assessed the proportion of alerts correctly issued when triggers were breached, and the proportion of false alerts issued when triggers were not breached.

### Relative change in health service utilisation

#### Data sources

We used the SAIL Databank and associated linkage methods to provide evaluation data.^15^ ^16^ The SAIL Databank contains anonymised linked demographic and health records for the population of Wales, including those derived from the NHS’ patient registration system, inpatient, outpatient, emergency department (ED) attendance and general practice data. Privacy protection is ensured by use of a split file, multiorganisation encryption design that removes all names, addresses, dates of birth and other identifiers within the NHS data and replaces them with unique non-identifying eight digit numbers for individuals and addresses before reassembling with the clinical content.^14^ The Patient Episode Dataset for Wales (PEDW) is held within SAIL and contains hospital admissions data recorded in Welsh hospitals. The SAIL Databank also contains data sets from the majority of GPs in Wales, including the four general practices from which patient lists were derived for this study. This is the first time an air pollution intervention has been evaluated using routine data linked at the individual level.

#### Variables

Our independent variable of interest was exposure to the airAware intervention. We derived health outcome measures from records using selected clinical READ codes related to respiratory conditions (asthma and COPD) and CHD (collectively referred to as relevant conditions). Outcome measures consisted of counts for: hospital admissions (emergency and elective, emergency only respiratory and CHD conditions combined and separately); ED attendances (non-trauma); outpatient attendances (non-trauma); primary care contacts (respiratory, CHD and mental health combined and separately) and prescribed medications (combined respiratory, CHD and mental health).

A hospital admission within PEDW was considered a relevant admission when any episode within that admission contained a primary diagnosis code for one of the relevant conditions, recorded using the International Classification of Diseases, 10th revision (ICD-10). Coding groups I20-I25, J42-J44 and J45 were used to identify records relating to CHD, COPD and asthma, respectively.

Primary care contacts were approximated by measuring the number of days on which an individual had any event recorded in the GP data (it is not possible to measure whether a patient visited the practice using the data provided to SAIL). Diagnoses, symptoms and treatments associated with a contact were identified using the Read code system V.2. The Read codes used to identify records related to the relevant conditions, as well as common mental health disorders, can be found in an online supplementary File.

We used the intervention groups’ sign-up date to the airAware system as their intervention start date. We randomly assigned pseudo sign-up dates to controls, matched to the distribution of participant sign-up dates, to derive comparable observation time periods for the intervention and control study arms. We collected preintervention data for up to 1 year for primary care outcomes and secondary care events (less frequent) for a 2-year period prior to the start of the intervention.

Dates of migration in and out of the study area and the date of death were both extracted from the databank for censoring purposes. We followed up all participants until: they moved out of the study area; they died; the end of the study period, whichever was earliest. We defined a participant's observation period end date of 30 April 2014 for primary care and 1 August 2014 for secondary care data.

#### Bias

Bias is possible in any evaluation of a non-randomised intervention. We expected to observe self-selection bias in a voluntary participation study such as this. We attempted to control for bias by using a controlled ‘before and after’ approach comparing change in health service utilisation between intervention and control arms. We adjusted for preintervention differences likely to influence the outcome of interest, such as age, gender, deprivation index and smoking status (current and past).

#### Study size

This pragmatic evaluation of an existing service used all available routinely collected patient data to determine health outcomes, and no formal power calculation was conducted.

### Statistical methods

#### System performance

We assessed the validity of the intervention by considering system sensitivity and specificity. In the context of this evaluation, sensitivity indicates the proportion of alerts that were correctly issued when system alert triggers were met and specificity is the proportion of alerts correctly not issued when system alert triggers were not met. We also assessed the positive predictive value (PPV), the proportion of all alerts sent that were true positives; and NPV, the proportion of all occasions when alerts were not required and not sent. To calculate system validity, the total assessment denominator was 13 140, defined as a 730-day (2 years) test period with potential for a maximum of three alerts per day from each of the six monitors.

#### Relative change in health service utilisation

We compared the preintervention characteristics of the intervention group with those of the control group; differences were tested for significance using ORs and χ^2^ tests. We compared health outcomes of the two study groups during the preintervention period with unadjusted and adjusted incidence rate ratios (IRRs). We also compared outcomes between the two groups in the postintervention period.

For our main analysis, we created separate, negative binomial regression models for each health service utilisation measure. We compared the total number of unique health utilisation counts for each participant in the time period before the sign-up/pseudo sign-up date, with the time period immediately after. The estimated change in healthcare utilisation associated with the intervention was derived from the interaction term between the participation group and the time period in the regression model.

We tested confounding variables for significant associations with the dependent variables and possible interaction effects were also explored. Random effects were included to account for dependence between premeasures/postmeasures for the same individuals and the natural logarithm of the number of observation days for each participant was added as an offset to account for the different lengths of data collection periods. We exponentiated coefficients of the regression model to produce adjusted IRR, in order to aid interpretation.

## Results

### System performance

Overall system validity is summarised for the entire pilot period in table 1. The sensitivity of the system was 83.9% (95% CI 78.8% to 87.9%) corresponding to a total of 208 PM_10_ alerts correctly issued (187 yellow alerts, 17 amber alerts and 4 red alerts) out of a total of 248 alerts that should have been issued. Specificity was 99.5% (95% CI 99.3% to 99.6%) relating to the proportion of alerts correctly not issued when system alert triggers were not met. A total of 67 out of 275 alerts sent were false positives, giving a PPV of 75.6% (95% CI 70.2% to 80.3%). The NPV was 99.7% (95% CI 99.6% to 99.8%)) relating to 12 825 true-negative events out of the 12 865 occasions where an alert was not sent.

**Table 1:** Validity of the alerts issued by the airAware system

	Alert trigger met		
	Yes	No	Total
Alert issued			
Yes	208	67	275
No	40	12 825	12 865
Total	248	12 892	13 140

### Participants

**Figure 2:**
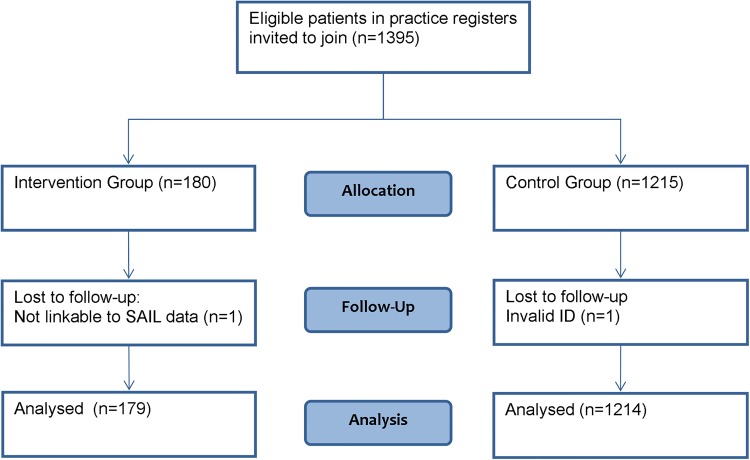
Flow of patients through airAware system and evaluation. SAIL, Secure Anonymised Information Linkage.

A total of 1395 patients attending the four practices met the inclusion criteria, and 180 signed up for the intervention. Two people, one in each of the intervention and control groups could not be linked to the data in SAIL and were excluded from analysis. Figure 2 shows the flow of patients in the study. We took into account the varying observation periods for each participant depending on their sign-up date. Recruitment took place over more than 1 year but the majority of participants (90.6%) signed up between May 2012 and July 2012, with the remainder signing up before April 2013; 98.4% had 2 years of data preintervention for secondary care outcomes; 89.8% had 1 year of data preintervention for primary care outcomes.

### Descriptive data

Preintervention characteristics showed the modal age group was 65–74 years for the intervention and control groups (table 2). There was a higher proportion of older participants in the intervention group compared with the control group, resulting in statistically significant differences in the age distributions (χ^2^=46.3, p<0.001). There were statistically non-significant differences by gender, deprivation and smoking status.

**Table 2:** Preintervention characteristics of the intervention and control groups

Sociodemographic characteristics	Intervention group	Control group	Total
Age			
0–14	7 (3.9%)	87 (7.2%)	94 (6.7%)
15–24	5 (2.8%)	99 (8.2%)	104 (7.5%)
25–34	9 (5.0%)	87 (7.2%)	96 (6.9%)
35–44	13 (7.3%)	107 (8.8%)	120 (8.6%)
45–54	14 (7.8%)	166 (13.7%)	180 (12.9%)
55–64	37 (20.7%)	187 (15.4%)	224 (16.1%)
65–74	60 (33.5%)	220 (18.1%)	280 (20.1%)
75–84	26 (14.5%)	172 (14.2%)	198 (14.2%)
85+	8 (4.5%)	89 (7.3%)	97 (7.0%)
Gender			
Female	92 (51.4%)	583 (48.0%)	675 (48.5%)
Males	87 (48.6%)	631 (52.0%)	718 (51.5%)
WIMD quintile			
Least deprived and next least deprived	16 (8.9%)	95 (7.8%)	111 (8.0%)
Average deprivation	31 (17.3%)	214 (17.6%)	245 (17.6%)
Next most deprived	106 (59.2%)	742 (61.1%)	848 (60.9%)
Most deprived	26 (14.5%)	163 (13.4%)	189 (13.6%)
Current smoker			
No	153 (85.5%)	1035 (85.3%)	1188 (85.3%)
Yes	26 (14.5%)	179 (14.7%)	205 (14.7%)
History of smoking			
No	90 (50.3%)	721 (59.4%)	811 (58.2%)
Yes	89 (49.7%)	493 (40.6%)	582 (41.8%)

WIMD, Welsh Index of Multiple Deprivation.

### Health outcome results

**Figure 3:**
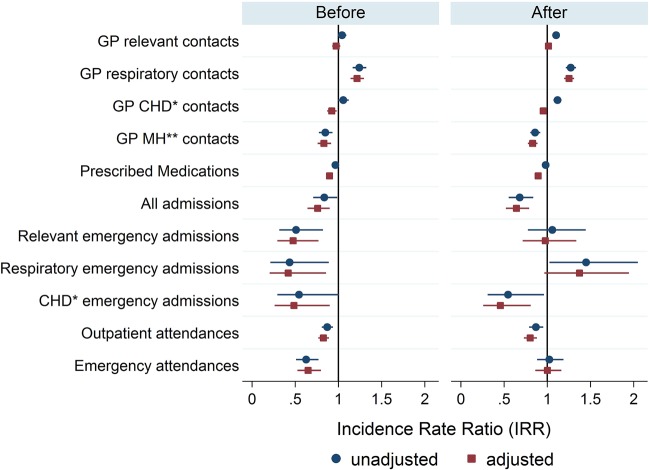
Differences between groups (unadjusted and adjusted IRR and 95% CIs) for preintervention and postintervention groups. An IRR >1 indicates a higher rate in the intervention group compared with the control group. CHD, coronary heart disease; GP, general practitioner; IRR, incidence rate ratio; MH, mental health.

Preintervention, the intervention group had fewer hospital admissions; all admissions, relevant emergency admissions, respiratory emergency admissions and CHD-related emergency admissions (figure 3). The intervention group had lower rates of outpatient attendances, ED attendances, GP contacts for mental health conditions, GP contacts for CHD conditions and slightly lower rates of prescriptions. Numbers for each outcome and rates both preintervention and postintervention are given in an online appendix table. The rate of GP contacts for respiratory conditions was higher among the intervention group compared with the control group. GP contacts for all relevant conditions were slightly higher for the intervention group but this was not statistically significant.

### Intervention effect results

Variables listed in table 2 have been established as potential confounders and were used to adjust the final model. The intervention was associated with significant increases in all relevant emergency admissions, respiratory-related emergency admissions and ED attendances (table 3).

**Table 3:** The intervention effect (IRR and 95% CI)

Outcome measure	Intervention effect	
	IRR	95% CI
GP relevant contacts	1.04	0.98 to 1.11
GP respiratory contacts	1.04	0.96 to 1.13
GP CHD contacts	1.02	0.95 to 1.11
GP MH contacts	0.98	0.84 to 1.16
Prescribed medications	1.03	0.98 to 1.09
All admissions	0.82	0.58 to 1.14
Relevant emergency admissions	**2.04**	**1.06 to 3.93**
Respiratory emergency admissions	**3.97**	**1.59 to 9.93**
CHD emergency admissions	0.97	0.39 to 2.42
Outpatient attendances	1.01	0.83 to 1.25
Emergency attendances	**1.89**	**1.34 to 2.68**

Statistically significant IRRs are in bold.CHD, coronary heart disease; GP, general practitioner; IRR, incidence rate ratio; MH, mental health.

### Additional analyses

We completed analyses to investigate if excess admissions occurred during periods of higher pollution by restricting observation periods to 7 days following PM_10_ low band exceedance. There were insufficient numbers (and in some cases no records) of secondary care outcomes for analysis. Estimates of effects were derived for GP outcomes, prescribed medications and outpatient visits. There was no statistically significant effect associated with the intervention when looking at events immediately following pollution episodes (results not shown).

## Discussion

Our objectives were to evaluate the accuracy of the airAware system in correctly identifying pollution episodes and issuing alerts; and evaluate the effect of the system on subsequent health service utilisation.

### Key results

The airAware air pollution alerting system missed a small proportion of alerts and issued 67 false alerts among 275 alerts issued. During the 2-year study period, system performance sensitivity was 83.9% and specificity was 99.5%. Participants with existing respiratory and CHD conditions who were in the intervention arm had a greater relative change in health service use following the start of the intervention compared with those in the control arm of the study. The intervention group experienced a doubling of emergency admissions for all relevant conditions and a fourfold admissions increase for respiratory conditions. These findings are important given one intention of these public health interventions is to reduce health service utilisation.

### Comparison with previous studies

This is the first study of an air pollution alert system reporting a statistically significant adverse difference in health service utilisation. This is noteworthy, given that several systems are in operation but have been unable to detect such an association, having been evaluated qualitatively, using self-report data, or quantitatively assessing changes within the total population using an ecological study design.^9^ ^10^ ^13^ Our study has several strengths. First, we used a search of disease registers in the local GPs to identify all those meeting the high-risk guidelines who would potentially benefit from the alerts. Second, using a data linkage system, we specifically flagged those who signed up to the intervention with their precise date of intervention start. Third, the use of retrospective and prospective data linkage is novel, because it allowed us to extract detailed anonymised healthcare utilisation for the control group in the same way as for the intervention group with almost no loss to follow-up and the avoidance of recall bias. Fourth, the use of six pollution monitors in a small area means that individual exposures and breaches of limits were more accurately measured than across entire cities.

### Limitations

This non-randomised observational study was conducted in the specific context of exposure in a small area. Evaluations undertaken in different settings may produce different results because the potential for benefits and harms from air alerting services will depend on local pollution profiles. It was not possible to randomise people into control and intervention arms of the study for this intervention. Participants were a self-selecting sample with different characteristics from the control population in terms of preintervention primary and secondary health services utilisation. The intervention group were characterised by their engagement in preventative health, as suggested from their relatively higher rate of GP events (figure 3) preintervention, resulting in a lower rate of emergency admissions compared with the control group preintervention.

Although we made multiple design and analysis adjustments to control for bias, it is likely that some residual confounding remains. Our use of a controlled pre-post regression analysis of health service utilisation, adjusting for socioeconomic characteristics recorded in routine data, will not have captured the impact of unmeasured confounders.

### Interpretation

The increase in emergency hospital admissions for signing up to the intervention was largely as a result of respiratory conditions. We suggest that the message to ‘follow your doctor's usual advice’ received by the intervention group, resulted in a heightened awareness in a group of people with chronic conditions who are unwell frequently. Patients did not increase GP activity but principally accessed EDs. Many of these patients will have chronic limitations in respiratory function which, in the context of reporting feeling unwell and being assessed by doctors unfamiliar with their usual status, may have resulted in admission to hospital. We propose that the alerts caused worry to the extent that it prompted intervention group participants to seek medical care and this in itself constitutes harm.^18^

### Generalisability

While all AQMAs have features that are unique and hence the potential trade off for benefits and harms associated with air pollution alerting services will not be the same in any two areas, aspects of this study may be generalisable. It is likely that the behaviour of people who have signed up to such services elsewhere are similar to those in this study and the same consequences of excess health service utilisation would arise. Replication is an important component of science and we would like to see this study repeated in another environment, particularly if randomising participants to the intervention group cannot be achieved.

### Conclusion

This study of a near real-time air pollution alert was associated with increased use of health services. Our findings raise questions about the trade off between harms and benefits of air pollution alerting services. This intervention was associated with an outcome that was in the opposite direction to expected from the COMEAP guidelines. There is a growing evidence base demonstrating some public health interventions are harmful.^19^ Wider roll-out of such systems does not appear to be warranted given the current evidence base.

## Other information

### Ethics and information governance

This study received approval from an independent Information Governance Review Panel (IGRP), an independent body consisting of membership from a range of government, regulatory and professional agencies.^15^ The cohort has also been assessed by the Multi-centre Research Ethics Committee for Wales and judged to be an anonymised research database which does not require ethical review in line with National Research Ethics Service guidance. In compliance with IGRP rulings, and in line with the Data Protection Act 1998,^20^ individual-level data and the corresponding encrypted linking field codes were not removed from the SAIL databank and thus were not included in exploratory or reference documents. Analyses were carried out within the SAIL Gateway, which provides a secure remote access service to the SAIL databank held at Swansea University Medical School.^21^

**What is already known on this subject**

- It is well established that poor air quality causes substantial excess mortality and morbidity and additional symptoms, particularly for high-risk individuals with existing respiratory and coronary heart disease.
- Government policy promotes advising high-risk individuals of imminent pollution incidents to encourage behaviour modification to reduce the impact of pollution.
- Evaluation of the effectiveness of air pollution alerting systems has been limited to small scale qualitative and self-report data and larger scale ecological study designs.

**What this study adds**

- A robust quantitative evaluation using longitudinal linked individual-level heath records to evaluate an air pollution alert intervention.
- Evidence for unanticipated harms through a statistically significant increase in health service utilisation in the intervention group.

## References

[1] DEFRA. *The Air Quality Strategy for England, Scotland, Wales and Northern Ireland (Volume 1)* 2007 Available at: https://www.gov.uk/

[2] Seaton A, Godden D, MacNee W, et al Particulate air pollution and acute health effects. Lancet 1995;345:176–8. 10.1016/S0140-6736(95)90173-6 7741860

[3] Dockery DW, Pope CA III, Xu X, et al An association between air pollution and mortality in six U.S. cities. N Engl J Med 1993;329:1753–9. 10.1056/NEJM199312093292401 8179653

[4] Pope CA III, Burnett RT, Thun MJ, et al Lung cancer, cardiopulmonary mortality, and long-term exposure to fine particulate air pollution. JAMA 2002;287:1132–41. 10.1001/jama.287.9.1132 11879110 PMC4037163

[5] Ayres J Review of the UK Air Quality Index. Committee on the Medical Effects of Air Pollutants 2011 Available at: http://www.comeap.org.uk

[6] Shah AS, Langrish JP, Nair H, et al Global association of air pollution and heart failure: a systematic review and meta-analysis. Lancet 2013;382:1039–48. 10.1016/S0140-6736(13)60898-3 23849322 PMC3809511

[7] Kelly FJ, Fuller GW, Walton HA, et al Monitoring air pollution: use of early warning systems for public health. Respirology 2012;17:7–19. 10.1111/j.1440-1843.2011.02065.x 21942967

[8] World Health Organisation. Review of evidence on health aspects of air pollution—REVIHAAP Project 2013 Available at: http://www.euro.who.int

[9] AirText. http://www.airtext.info/ (accessed 3 Nov 2015).

[10] airAlert: air quality early warning service. http://www.airalert.info. http://www.airalert.info/Splash.aspx (accessed 3 Nov 2015)

[11] Know & Respond—Scotland, the free air pollution alert messaging system—Air Quality in Scotland. http://www.scottishairquality.co.uk/know-and-respond/ (accessed 3 Nov 2015)

[12] Met Office. Healthy Outlook®—helping patients with COPD this winter. | Met Office News Blog. http://blog.metoffice.gov.uk/2012/11/01/healthy-outlook-helping-patients-with-copd-this-winter/ (accessed 3 Nov 2015)

[13] Walton H, Baker T, Fuller G, et al Air pollution alert services evidence development strategy—prediction of possible effectiveness and assessment of intervention study feasibility. Vol 83 King's College London, 2014.

[14] Lyons RA, Jones KH, John G, et al The SAIL databank: linking multiple health and social care datasets. BMC Med Inform Decis Mak 2009;9:3 10.1186/1472-6947-9-3 19149883 PMC2648953

[15] Ford DV, Jones KH, Verplancke JP, et al The SAIL Databank: building a national architecture for e-health research and evaluation. BMC Health Serv Res 2009;9:157 10.1186/1472-6963-9-157 19732426 PMC2744675

[16] Lyons RA, Ford DV, Moore L, et al Use of data linkage to measure the population health effect of non-health-care interventions. Lancet 2014;383:1517–19. 10.1016/S0140-6736(13)61750-X 24290768

[17] GMS Contract—QOF Business Rules 2014/15 Version 30. http://www.wales.nhs.uk/sites3/page.cfm?orgid=480&pid=76672 (accessed 8 May 2015)

[18] Maughan D, Ansell J Protecting resources, promoting value: a doctor's guide to cutting waste in clinical care. Vol 60 Academy of Medical Royal Colleges, 2014.

[19] Bonell C, Jamal F, Melendez-Torres GJ, et al ‘Dark logic’: theorising the harmful consequences of public health interventions. J Epidemiol Community Health 2015;69:95–8. doi:10.1136/jech-2014-204671 10.1136/jech-2014-204671 25403381

[20] Data Protection Act 1998 1998 Available at: http://legislation.gov.uk

[21] Jones KH, Ford DV, Jones C, et al A case study of the Secure Anonymous Information Linkage (SAIL) Gateway: a privacy-protecting remote access system for health-related research and evaluation. J Biomed Inform 2014;50:196–204. 10.1016/j.jbi.2014.01.003 24440148 PMC4139270

