# Supplementary material for: Effects of an air pollution personal alert system on health service usage in a high-risk general population: a quasi-experimental study using linked data
Source: J Epidemiol Community Health. 2016 May 23;70(12):1184–90. doi: 10.1136/jech-2016-207222 (PMC5136690; doi:10.1136/jech-2016-207222)
Supplement: Supplementary data [file jech-2016-207222supp.pdf]

Insert Practice Header

airAware Port Talbot

Air Quality and Health Project

Do you want to be part of a new project that may help protect your health?

Research shows that local air quality can have an impact on people's health, especially those who have a chronic heart or lung condition. Monitoring of the air in Port Talbot shows that it has improved over many years. There remain however, short periods of time when air quality fails to meet normal standards.

New technology has allowed us to develop an air quality information system called "airAware". This will automatically send you a message to let you know when air quality may affect your health, and give you timely advice on actions you could take to reduce possible health effects.

The system will be tested in an area of Port Talbot where the air quality is measured continuously. We would like to invite you to join this free service. You can choose to receive the health advice messages via e-mail, text (SMS) to a mobile telephone or a recorded voice message to a landline.

The project will run for up to two years and we will be looking at feedback from you to see whether it would be useful to make the service more widely available. Your help would be invaluable.

If you think this service might be of interest to you, please read the enclosed information on how to sign up, or if you have internet access, look at our website www.airaware.co.uk

If you would like more information regarding this service, please contact the dedicated helpline based at NHS Direct Wales on 0300 100 1300. They will be happy to help.

With best wishes,

Dr Sanjay Challisery, Afan Community Network Lead, Neath Port Talbot Locality, ABM Health Board

Dr Ann Delahunty, Public Health Consultant, Public Health Wales

Welcome to airAware Port Talbot

This leaflet contains information about how airAware works and advice on what you can do when you receive an airAware message

Croeso i airAware Port Talbot

Mae'r daflen hon yn cynnwys gwybodaeth am sut mae airAware yn gweithio a chyngor ar yr hyn y gallwch ei wneud pan fyddwch yn derbyn neges airAware

Welcome to airAware!
You are invited to sign up to airAware, a unique air quality information service for people in Port Talbot who suffer from heart or lung conditions including asthma. If you have one of these conditions you may be affected by the quality of air around you.

airAware will alert you to air quality that may affect your health so that you can take simple steps to help reduce the likelihood of any effects. This includes taking your inhaler or angina spray with you, taking extra doses if symptoms worsen (as previously advised by your doctor or nurse) and avoiding strenuous outdoor activity on those days. If you care for someone with these symptoms it may help you look after them better.

The service is based upon advice from The Committee on the Medical Effects of Air Pollutants (COMEAP) on air quality.

Croeso i airAware!
Fe'ch gwahoddir i gofrestru gydag airAware, sef gwasanaeth gwybodaeth unigryw am ansawdd aer i bobl ym Mhort Talbot sy'n dioddef o gyflwr ar y galon neu'r ysgyfaint gan gynnwys asthma. Os oes gennych un o'r cyflyrau hyn, gallai ansawdd yr aer o'ch cwmpas effeithio arnoch chi.

Bydd airAware yn eich rhybuddio am ansawdd aer a allai effeithio ar eich iechyd fel y gallwch gymryd camau syml i helpu i leihau'r tebygolrwydd y bydd unrhyw effeithiau. Gallai hyn gynnwys cludo'ch mewnanadlydd neu eich chwistrelliad angina gyda chi, cymryd dognau ychwanegol os bydd y symptomau'n gwaethygu (os yw eich meddyg neu eich nyrs wedi'ch cynghori i wneud hyn ymlaen llaw) ac osgoi gweithgareddau awyr agored ar y diwrnodau hynny. Os ydych yn gofalu am rywun sydd ag unrhyw un o'r symptomau hyn, gallai eich helpu i ofalu amdano'n well.

Mae'r gwasanaeth yn seiliedig ar gyngor gan Pwyllgor Effeithiau Meddygol ar Lygryddion Aer ar ansawdd aer (COMEAP).

Air Quality and Health

COMEAP advises that poor air quality can worsen the condition of individuals with heart or lung disease and can aggravate, but not cause asthma.

Healthy Individuals

Are unlikely to notice or suffer from any serious or lasting ill-health effects from levels of air quality experienced in the UK.

People with asthma or lung disease

Symptoms are unlikely to change when air quality levels are in the green band. This applies whatever the time of year. Symptoms may get worse as air quality deteriorates, especially for the elderly. Steps should then be taken to prevent or reverse the effects of air pollutants as with other triggers of asthma attacks such as cold air and exercise. You may need to take your inhalers more frequently. People with angina may need to use their nitrate spray.

Follow your doctor's usual advice about managing your condition. If these steps don't help, seek medical advice as you usually would.

PORT TALBOT RESOURCE CENTRE

GIG CYMRU NHS WALES Bwrdd Iechyd Prifysgol Abertawe Bro Morgannwg University Health Board

Health Protection Agency

Ansawdd Aer ac Iechyd

Mae COMEAP yn nodi y gall ansawdd aer gwael waethygu cyflwr unigolion ag afiechydon y galon neu'r ysgyfaint ac y gall waethygu, ond nid achosi, asthma.

Unigolion Iachus

Yn annhebygol o sylwi neu ddioddef unrhyw effeithiau difrifol neu barhaus o lefelau ansawdd aer a brofir yn gyffredin yn y DU.

Pobl yn dioddef o asthma neu cyflwr yr ysgyfaint

Mae symptomau yn anhebygol o newid pan mae lefel yr ansawdd aer yn y band werdd. Mae hwn yn wir trwy gydol y flwyddyn. Gall symptomau gwaethygu pan mae ansawdd aer yn dirywio, yn enwedig yn yr henoed. Dylid cymryd camau i atal neu gwrthdroi effeithiau llygredd aer fel mae gwneud gyda achosion asthma fel gweithgareddau yn yr awyr agored. Efallai bydd angen defnyddio'ch mewnanadlydd yn fwy cyson. Efallai bydd angen nitrate spray ar bobl yn dioddef o angina.

Dilynnwch cyngor arferol eich meddyg am reoli eich cyflwr. Os nad yw'r camau yma yn helpu dylech geisio cyngor meddygol yn ôl yr arfer.

PORT TALBOT RESOURCE CENTRE

GIG CYMRU NHS WALES Bwrdd Iechyd Prifysgol Abertawe Bro Morgannwg University Health Board

Asiantaeth Diogelu Iechyd

Health Message

The following health messages will be sent to you when the air quality level reaches the yellow band or above.

Air Quality Band	Public Health Message (Messages based on COMEAP advice)
**Green**	Enjoy your usual outdoor activities
**Yellow**	If you experience symptoms **consider reducing** strenuous physical activity, particularly outdoors
**Amber**	**Reduce** strenuous physical activity, particularly outdoors. Keep reliever inhaler with you. Follow doctor's usual advice about managing your condition
**Red**	**Avoid** strenuous physical activity, particularly outdoors. Keep reliever inhaler with you. Follow doctor's usual advice about managing your condition

Neges Iechyd

Caiff y negeseuon iechyd canlynol eu hanfon atoch pan fydd lefelau ansawdd yr aer yn cyrraedd y band melyn neu'n uwch.

Band Ansawdd yr Aer	Neges Iechyd Cyhoeddus (yn seiliedig ar gyngor COMEAP)
**Gwyrdd**	Dylech fwynhau eich gweithgareddau awyr agored arferol
**Melyn**	Os ydych yn profi symptomau, **dylech ystyried lleihau** gweithgareddau corfforol sy'n peri straen, yn enwedig yn yr awyr agored
**Oren**	**Dylech leihau** gweithgareddau corfforol sy'n peri straen, yn enwedig yn yr awyr agored. Dylech gludo eich mewnanadlydd gyda chi. Dilynwch gyngor arferol eich meddyg am reoli eich cyflwr
**Coch**	**Dylech osgoi** gweithgareddau corfforol sy'n peri straen, yn enwedig yn yr awyr agored. Dylech gludo eich mewnanadlydd gyda chi. Dilynwch gyngor arferol eich meddyg am reoli eich cyflwr

How does airAware work?
When air quality may affect your health, you will receive a health advice message. This automated service will operate between 7am and 10pm. Outside of these hours information will be available at www.airaware.co.uk

You can receive a message by selecting ONE of the following:

Mobile phone
airAware will send a text message to your (or your carer's) mobile phone. Messages will give air quality information and brief health advice.

or
Voice message (to home phone)
Your home phone will ring as normal and you will hear a recorded message. airAware can leave a message on your answer phone (if you have one), just like any other message.

or
e- mail
You will receive air quality information and brief health advice via email to your selected address.

Sut mae airAware yn gweithio?
Pan fo'n bosib y bydd ansawdd yr aer yn effeithio ar eich iechyd, byddwch yn derbyn neges o gyngor. Bydd y gwasanaeth awtomatig hwn ar waith rhwng 7am a 10pm. Y tu allan i'r oriau hyn, bydd gwybodaeth ar gael yn www.airaware.co.uk

Gallwch dderbyn neges drwy ddewis UN o'r canlynol:

Neges Ffôn Symudol
Bydd airAware yn anfon neges destun at eich ffôn symudol chi (neu eich gofalwr). Bydd y negeseuon yn rhoi gwybodaeth am ansawdd yr aer a chyngor bras am iechyd.

neu
Neges lais (i ffôn eich cartref)
Bydd airAware yn ffonio yn ôl yr arfer a byddwch yn clywed neges wedi'i recordio. Bydd airAware yn gadael neges ar eich peiriant ateb (os oes gennych un), fel unrhyw neges arall.

neu
e-bost
Byddwch yn derbyn yr un wybodaeth am ansawdd yr aer a gwybodaeth a chyngor bras ar iechyd drwy e-bost i'r cyfeiriad dewisiwyd.

What should I do when I receive an airAware alert?
The messages will provide you with advice depending on measured levels of air quality and the various simple steps you can take to reduce its effect on you.

How often will I receive an airAware message?
We have divided air quality into 4 bands. We will send a message whenever measured air quality does not meet the green band. Messages are only sent out between 7am and 10pm. You will receive further messages if air quality gets worse and the following day you will receive an update at 7am.

What happens if I change my phone number or email address?
You can update *any* of your personal (or your carer's) details at www.airaware.co.uk If you do not have internet access please call the Neath Port Talbot Council call centre on 01639 686868 and ask for the airAware helpline.

Beth y dylwn i ei wneud pan fyddaf yn derbyn neges gan airAware?
Bydd y neges yn rhoi cyngor i chi gan ddibynnu ar lefelau ansawdd yr aer a fesurwyd a'r camau syml y gallwch eu gwneud i leihau'r effaith arnoch chi.

Pa mor aml y byddaf yn derbyn neges airAware?
Rydym wedi rhannu ansawdd yr aer yn 4 band. Byddwn yn anfon neges pan fydd ansawdd yr aer a fesurwyd ddim yn cyrraedd y band gwyrdd). Anfonir negeseuon rhwng 7am a 10pm yn unig. Byddwch yn derbyn mwy o negeseuon os bydd ansawdd yr aer yn gwaethygu. Byddwch yn derbyn y newyddion diweddaraf y diwrnod canlynol am 7am.

Beth sy'n digwydd os ydw i'n newid fy rhif ffôn neu fy nghyfeiriad e-bost?
Gallwch ddiweddaru *unrhyw* fanylion personol (neu fanylion eich gofalwr) ar y safle www.airaware.co.uk Os nad oes gennych fynediad i'r rhyngrwyd, dylech ffonio canolfan galw Cyngor Castell Nedd Port Talbot ar 01639 86868 a gofyn am linell gymorth airAware.

What about when I go on holiday?
If you are going away or don’t want to receive the airAware messages for a while, you can log on to www.airaware.co.uk and suspend your messages.

If you do not have internet access please call the Neath Port Talbot Council call centre on 01639 686868 and ask for the airAware helpline.

Although UK mobile providers will not charge for receiving SMS/Text messages whilst abroad in the EU, it is possible for a user to suspend the receipt of SMS/Text messages during prolonged periods.

If I decide I no longer wish to receive alerts?
To stop receiving airAware messages, log onto www.airaware.co.uk If you do not have internet access please call the Neath Port Talbot Council call centre on 01639 686868 and ask for the airAware helpline.

Beth sy’n digwydd pan fyddaf ar wyliau?
Os ydych yn mynd i ffwrdd neu os nad ydych am dderbyn negeseuon gan airAware am gyfnod, gallwch fewngofnodi i’r safle www.airaware.co.uk a byddwn yn atal eich negeseuon airAware.

Os nad oes gennych fynediad i’r rhyngrwyd, dylech ffonio canolfan galw Cyngor Castell Nedd Port Talbot ar 01639 686868 a gofyn am linell gymorth airAware.

Er nad yw darparwyr ffonau symudol yn codi tal am dderbyn negeseuon destun yn yr UE, mae’n bosib trefnu peidio derbyn negeseuon yn ystod cyfnodau hir tramor.

Beth os ydw i’n penderfynu nad yw i am dderbyn negeseuon bellach?
Er mwyn stopio derbyn negeseuon airAware, dylech fewngofnodi i’r safle www.airaware.co.uk Os nad oes gennych fynediad i’r rhyngrwyd, dylech ffonio canolfan galw Cyngor Castell Nedd Port Talbot ar 01639 686868 a gofyn am linell gymorth airAware.

Do I need to do anything else?
As we are testing the service, you may be contacted to ask how useful you find the service and how we could make it better. If you do not wish to be contacted in this way, please let us know.

Is there anything else I need to know?
Air quality has significantly improved over the past 10 years and air quality is usually good. Most of the time even sensitive individuals will not notice any effect of poor air quality on their health.

When you receive an airAware message there is no need to become alarmed. Just be prepared, reduce physical activity, particularly outdoors, where possible and make sure that you carry any necessary medication. For example, if you have asthma, keep your reliever inhaler with you. Never exceed the stated dose of medication and never take medication that has been prescribed for someone else. If you feel unusually unwell you should seek medical advice as you normally would.

A oes angen i mi wneud unrhyw beth arall?
Oherwydd ein bod yn profi’r gwasanaeth, efallai y byddwn yn cysylltu â chi i ofyn pa mor ddefnyddiol y mae’r gwasanaeth a sut gallwn wella’r gwasanaeth. Os nad ydych am i ni gysylltu â chi yn y modd hwn, dywedwch wrthym.

A oes unrhyw beth y mae angen i mi ei wybod?
Mae ansawdd yr aer wedi gwella’n sylweddol dros y 10 mlynedd diwethaf ac fel arfer, mae ansawdd yr aer yn dda. Gan amlaf, nid fydd hyd yn oed unigolion sensitif yn sylwi ar unrhyw effaith ansawdd aer gwael ar eu hiechyd.

Pan fyddwch yn derbyn neges airAware, nid oes angen poeni. Byddwch yn barod, dylech leihau eich gweithgareddau corfforol, yn enwedig yn yr awyr agored a, lle y bo’n bosib, sicrhewch eich bod yn cymryd eich meddyginiaeth. Er enghraifft, os oes asthma arnoch chi, dylech gludo eich mewnanadlydd gyda chi. Peidiwch byth â chymryd gorddos o feddyginiaeth a pheidiwch byth â chymryd meddyginiaeth sydd ar gyfer rhywu arall. Os ydych yn teimlo’n anarferol o sâl, dylech geisio cyngor meddygol yn ôl yr arfer.

Port Talbot airAware Information System

You have been invited to sign up to the pilot Port Talbot airAware Information service. Full details of the service and background information have been provided in the leaflet and further information is also available on the website at **www.airaware.co.uk**

If you wish to register to take part in the pilot study, it is preferable to register online as this will give you the flexibility to change the service you receive as and when you wish.

Please register at:

www.airaware.co.uk/account/register

If you are unable to register online and wish to receive automated airAware messages by email, text or phone, please fill in the form on the reverse side of this page and return it in the prepaid envelope to:

Christopher Conolly (airAware Registration)
AEA
The Gemini Building
Fermi Avenue
Didcot Oxon
OX11 OQR

Sign up to airAware

Internal use only:
UN______________PW____________

Patient Details

Name			
Surname			
Gender		Date of Birth	/ /
Address			

Reference Code (optional)

Provided at the top of the letter inviting you to join the service

Reference Code	

Site Locations

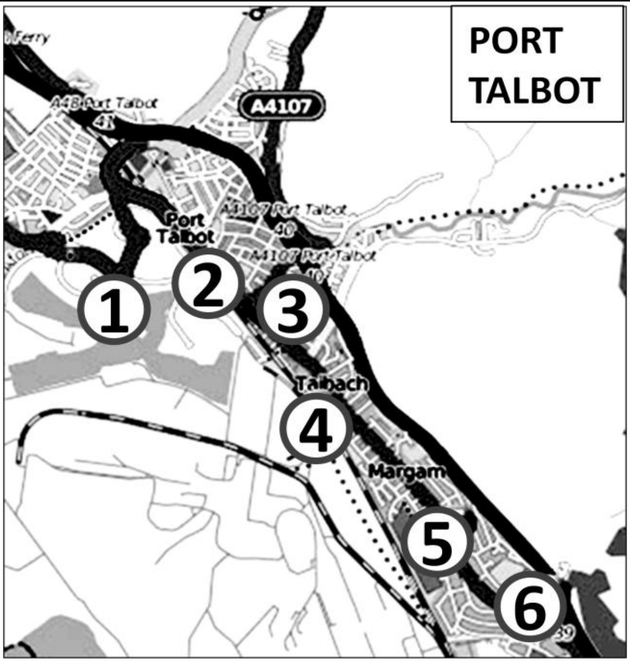

There are six monitoring sites in the area, please select the site nearest to where you spend most of your time between 7am and 10pm:

Tick One Only

- ☐ 1. Port Talbot Docks
- ☐ 2. Port Talbot, Talbot Road
- ☐ 3. Port Talbot, Theodore Road
- ☐ 4. Port Talbot, Margam (Fire Station)
- ☐ 5. Port Talbot, Twll-yn-y-Wal Park
- ☐ 6. Port Talbot, Dyffryn School

How would you like to receive your messages?

Messages will only be sent between 7am and 10pm (only one can be selected)

☐ **Email**	Email:
☐ **Text/SMS**	Mobile Phone number:
☐ **Phone (landline only)**	Phone number:

Submission

☐ **(required)**	I accept the terms and conditions of this alert system
☐ **yes** ☐ **No**	A small number of participants may be contacted by Swansea University to tell them about their experiences of this pilot service. Are you happy to be contacted?

Terms and Conditions:

By subscribing to the airAware Port Talbot information service you agree to the following terms and conditions of the service:

Subscribers are responsible for keeping their subscription details up to date. The service may be reviewed on a regular basis and it is planned to continue until the end of December 2013. To stop receiving messages, visit www.airaware.co.uk and deselect the relevant alerts through the 'Delivery Details' in the 'My Account' section and press the 'Save changes' button. Alternatively, contact Neath Port Talbot Council call centre on **01639 686868**.

The service may be ceased at anytime without notice or reason. Any personal contact data provided will only be used for the purposes of providing the service and will not be disclosed to anyone else, other than any disclosures required by law.

Limitations of the Service:

The air quality information presented in this system is based on hourly measurement data and no assurance can be given that this is 100% accurate as data will not be fully ratified at such short time interval between collection and a message being sent. The air quality level and public health messages are provided for guidance only. None of the organisations associated with the operation of the site accept any liability for actions arising from the decisions you make and/or the actions you take in response to any messages. AEA will use reasonable endeavours to provide alerts, but none of the organisations associated with the operation of the site accept any liability for failure to provide forecasts/alerts, or any liability for actions arising from any decisions you make and/or actions you take as a result of such failure.

Appendix Table: Health service utilisation pre-intervention and post-intervention (number and rate per person year)

	Pre-Intervention				Post-Intervention			
Health Service Utilisation	Intervention Group		Control Group		Intervention Group		Control Group	
	number	rate	number	rate	number	rate	number	rate
GP relevant contacts	1,939	10.85	3,854	12.21	23,153	11.08	12,504	10.42
GP respiratory contacts	1,149	6.43	2,233	7.08	11,614	5.56	6,215	5.18
GP CHD* contacts	1,300	7.27	2,623	8.31	15,520	7.43	8,268	6.89
GP MH** contacts	498	2.79	961	3.05	7,405	3.54	3,942	3.28
Prescribed Medications	11,016	61.63	21,752	68.93	146,631	70.19	76,622	63.83
All admissions	156	0.44	99	0.26	983	0.38	1,256	0.52
Relevant emergency admissions	18	0.05	46	0.12	294	0.11	239	0.10
Respiratory emergency admissions	8	0.02	39	0.10	182	0.07	125	0.05
CHD* emergency admissions	11	0.03	13	0.03	161	0.06	136	0.06
Outpatient attendances	797	2.23	500	1.31	3,897	1.50	6,170	2.57
Emergency attendances	98	0.27	201	0.52	1,328	0.51	1,056	0.44

*Coronary Heart Disease; **Mental Health
